# Dataset for influence of visual and haptic feedback on the detection of threshold forces in a surgical grasping task

**DOI:** 10.1016/j.dib.2022.108045

**Published:** 2022-03-11

**Authors:** John-John Cabibihan, Ahmad Yaser Alhaddad, Tauseef Gulrez, W. Jong Yoon

**Affiliations:** a Department of Mechanical and Industrial Engineering, Qatar University, Doha, Qatar; b Australian Public Service, Port Melbourne, Victoria, Australia; c School of Science, Technology, Engineering, and Mathematics, University of Washington, Bothell, WA, USA

**Keywords:** Haptics, Robotic surgery, Minimal force threshold, Surgical grasping

## Abstract

The data is related to minimal force thresholds perception in robotic surgical grasping applications. The experimental setup included an indenter-based haptic device acting on the fingertip of a participant and a visual system that displays grasping tasks by a surgical grasper. The experiments included the display of two presentations at different force levels (i.e., grasping and indentation) in three different modes, namely, visual-alone, haptic-alone, and bimodal (i.e., combined). For each mode, the participants were asked to identify which of the two presentations was higher. Each experiment was repeated till the termination conditions were met. Sixty participants took part in these experiments. The experiments were randomized and the threshold forces were calculated based on an algorthim. The datasets contain the individual responses of each participant, the threshold forces calculations, and the number of iterations.

## Specifications Table

**Table:** 

Subject	Biomedical engineering
Specific subject area	Robotic surgery
Type of data	Table
How data were acquired	The data were acquired from the responses of sixty participants to three modes of experiments related to force threshold perception in robotic surgery.
Data format	Raw and Analyzed (Force values)
Parameters for data collection	The conditions considered were the three different experimental modes. The considered termination conditions were the total number of iterations (i.e., 50) and the number of reversals (i.e., 8) based on the responses.
Description of data collection	The data were collected using a computer script that stored the responses of sixty participants during the experiments. Each participant has used the experimental setup to perform the visual-alone, haptic-alone, and bi-modal tests. After each experiment, the responses of each participant were stored locally.
Data source location	Institution: Qatar University City/Town/Region: Doha Country: Qatar
Data accessibility	Repository name: Harvard Dataverse Data identification number (permanent identifier, i.e. DOI number): 10.7910/DVN/QGKEUW Direct URL to data: https://dataverse.harvard.edu/dataset.xhtml?persistentId=doi:10.7910/DVN/QGKEUW
Related research article	J. -J. Cabibihan, A. Y. Alhaddad, T. Gulrez and W. J. Yoon, Influence of Visual and Haptic Feedback on the Detection of Threshold Forces in a Surgical Grasping Task, IEEE Robotics and Automation Letters. Vol. 6, no. 3, pp. 5525-5532, (2021). 10.1109/LRA.2021.3068934.

## Value of the Data

• The data characterize the smallest noticeable difference between different presentations in a surgical grasping task.
• The data are useful in identifying force threshold perception in robotic surgery.
• The data are useful for those working on the development of haptic devices for robotic surgery applications.
• The data can be used in the future evaluation of haptic feedback systems in similar applications.

## 1 Data Description

**Fig. 1:**
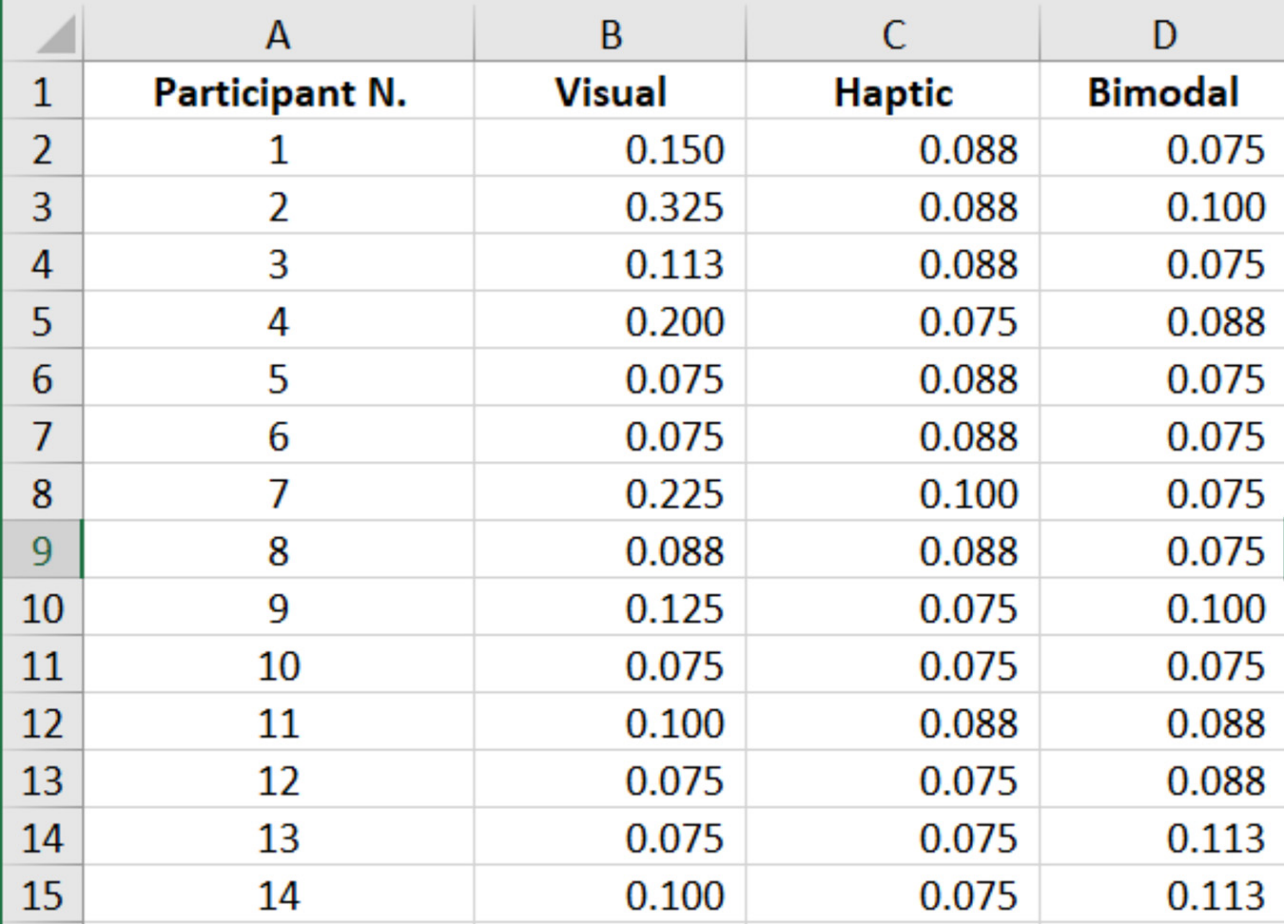
A screenshot of the content in file *“0_thresholdForce_analysis”* showing the participant number and the corresponding average threshold force in (N) achieved in the visual, haptic, and bimodal experiments.

**Fig. 2:**
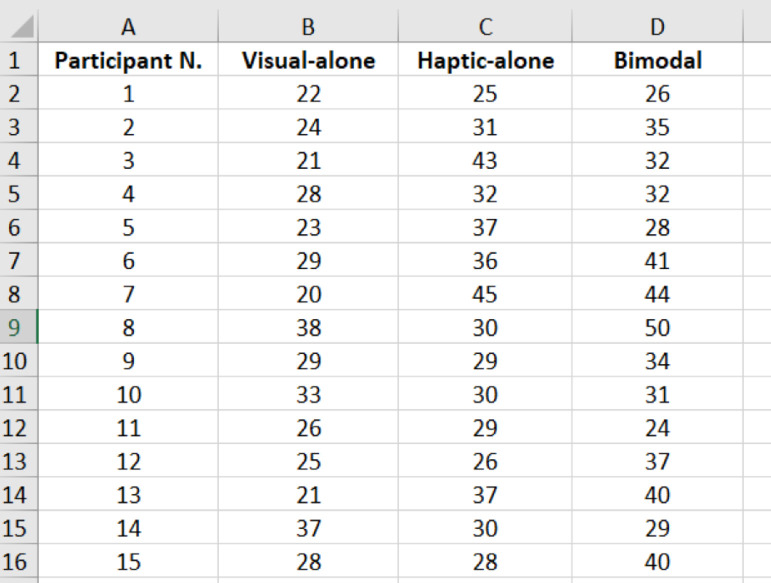
A screenshot of the content in file *“0_presentations_analysis”* showing the participant number and the corresponding iterations to achieve their threshold forces in the visual, haptic, and bimodal experiments.

**Fig. 3:**
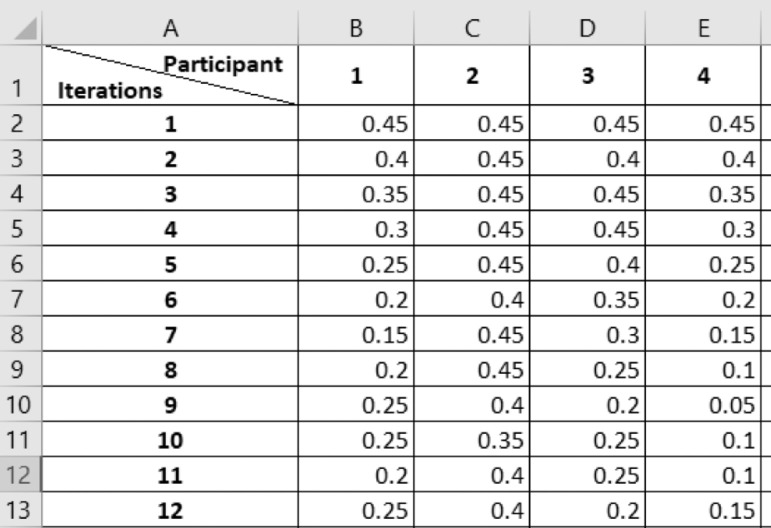
A screenshot of the content in file *“Visual-alone”* showing the participant number and corresponding changes in the perceived threshold force in (N) over the iterations.

The data link contains six datasets in excel format [1]. The datasets contain the responses of sixty participants to different presentations displaying different force levels in three different modes. The experimental modes considered were visual-alone feedback, haptic-alone feedback, and combined feedback. The file *“0_thresholdForce_analysis”* contains the average threshold force calculation in (N) of each participant for the three experiments (Fig. 1). The file *“0_presentations_analysis”* contains the iterations executed by each participant to reach their respective threshold forces (Fig. 2). Both of these files also contain the mean, standard deviation, and median for all the participants. The next three files “*Visual-alone”,” Haptics-alone*”, and “*Bimodal*”, contain the force threshold changes over iterations for the three experiments (Fig. 3). Each dataset also contains the mean threshold forces for every iteration across all participants in the last column. The file “*meanThresholdForce_last_four_reversals*” contains the threshold force values at the last four reversals. These values were used to calculate the mean and median values. This file contains three tabs for the three modes.

**Fig. 4:**
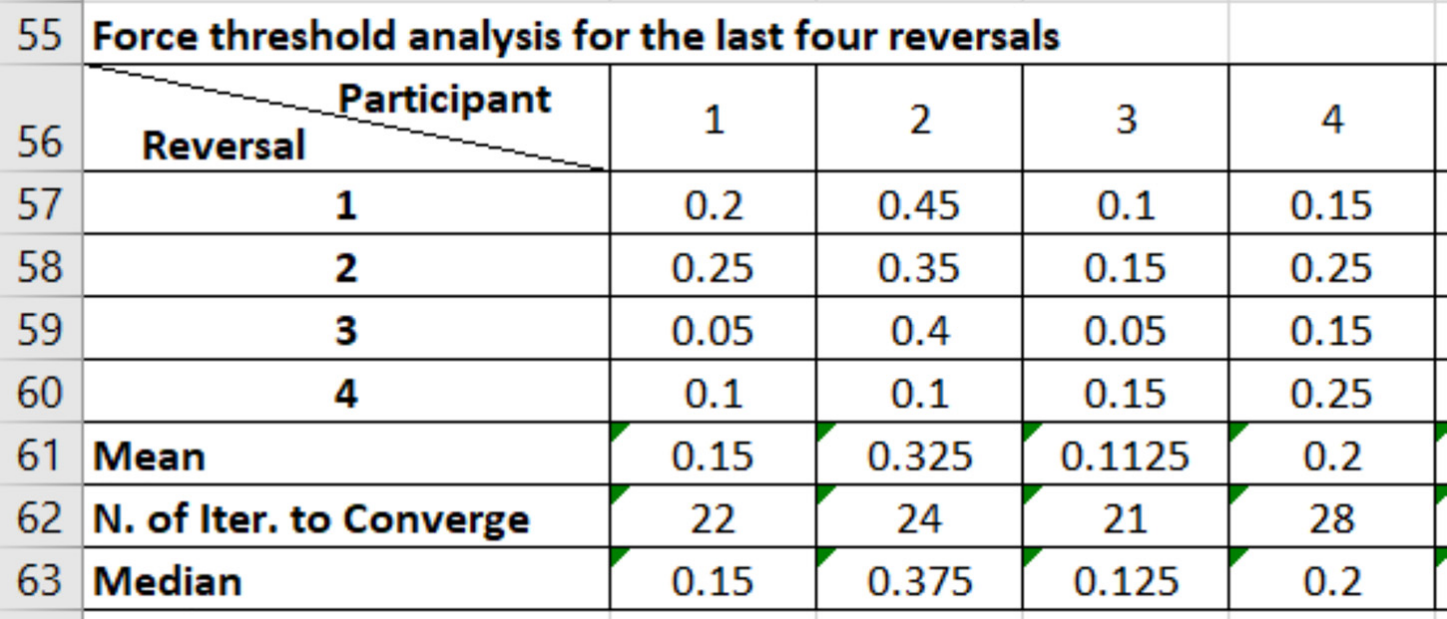
A screenshot of the content in file *“meanThresholdForce_last_four_reversals”* showing the mean and median in (N) for the last four reversals. It also shows the number of iterations took to complete an experiment.

## 2 Experimental Design, Materials and Methods

### 2.1 Experimental setup

**Fig. 5:**
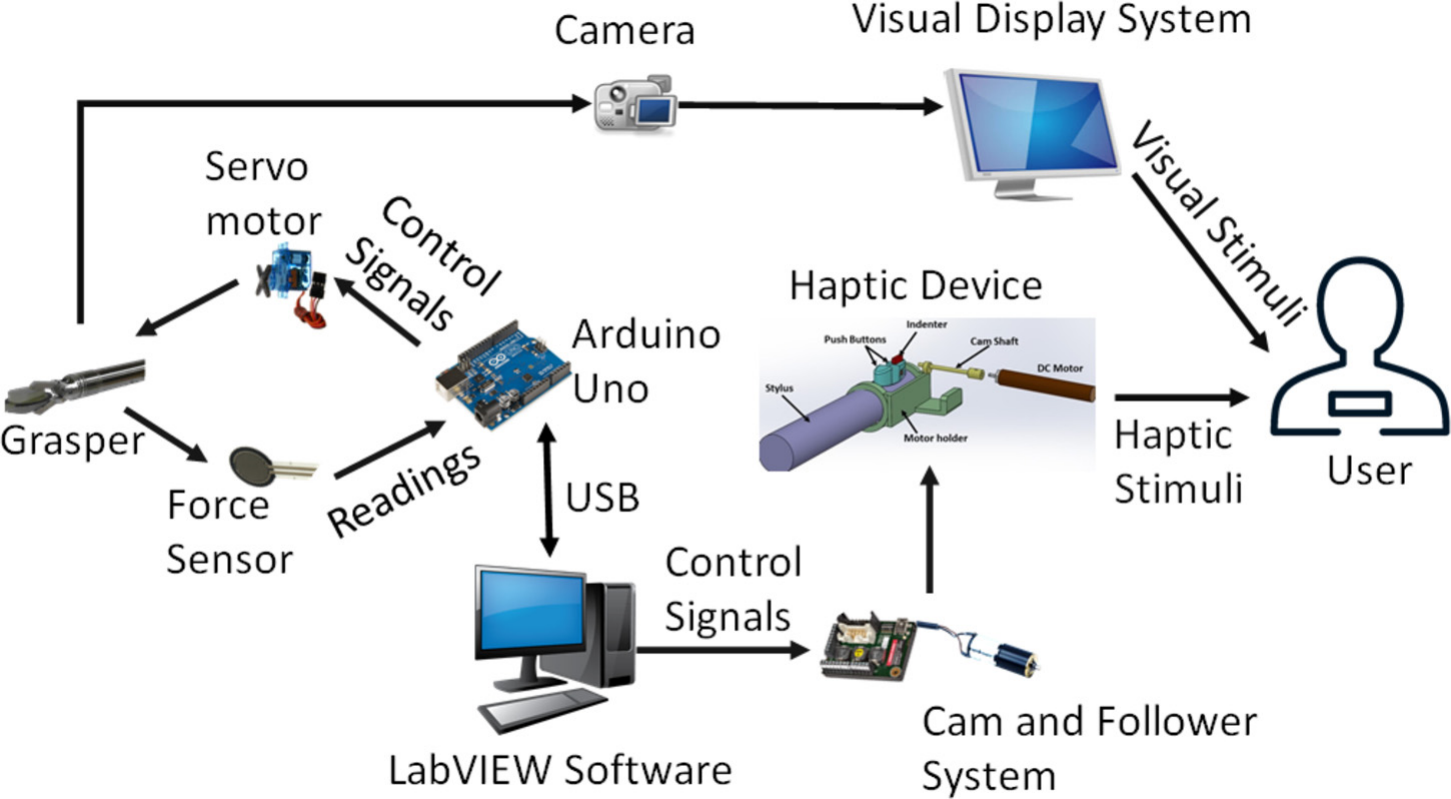
An overview of the experimental setup (Adapted from [2]).

The developed setup provided the visual and haptic feedback to the participants (Fig. 5). An indenter was developed to achieve the haptic-alone feedback by providing different indentations to a participant's figertip. A screen displaying different grasping levels provided the visual-alone feedback.

**Fig. 6:**
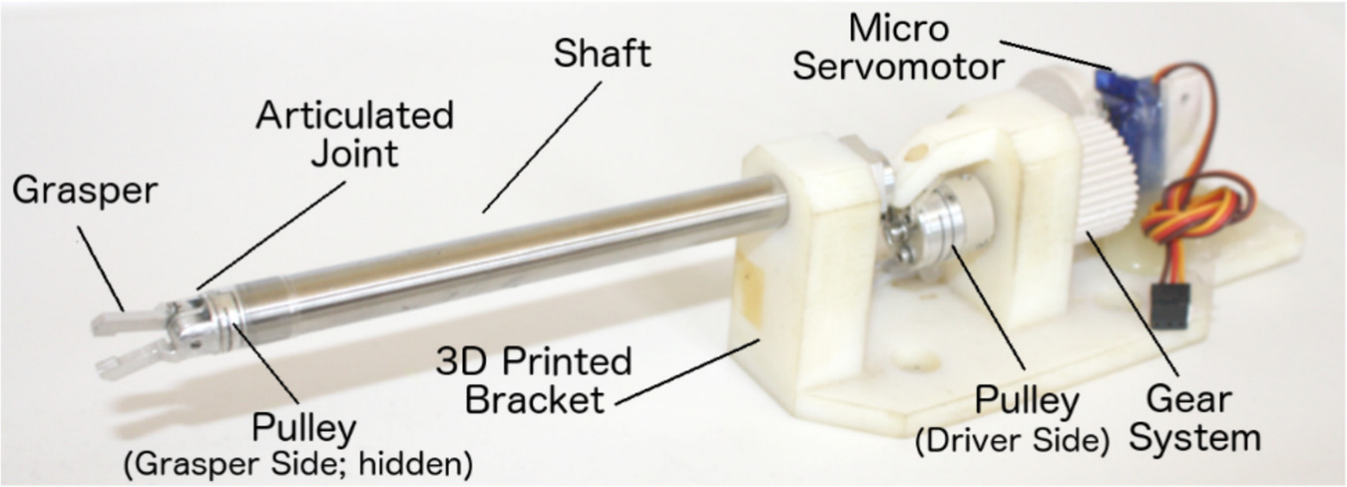
The surgical grasper that was used in the grasping tasks (Adapted from [2]).

Surgical grasper was used to generate the grasping tasks’ videos as part of the visual feedback in the experiments (Fig. 6). A servo motor was used to control the operations of the graspe through a cable-driven mechanism. An Arduino microcontroller was used to control the servo motor using a LabView script. A force sensor embedded in a soft tissue was used to measure the grasping forces of the grasper.

**Fig. 7:**
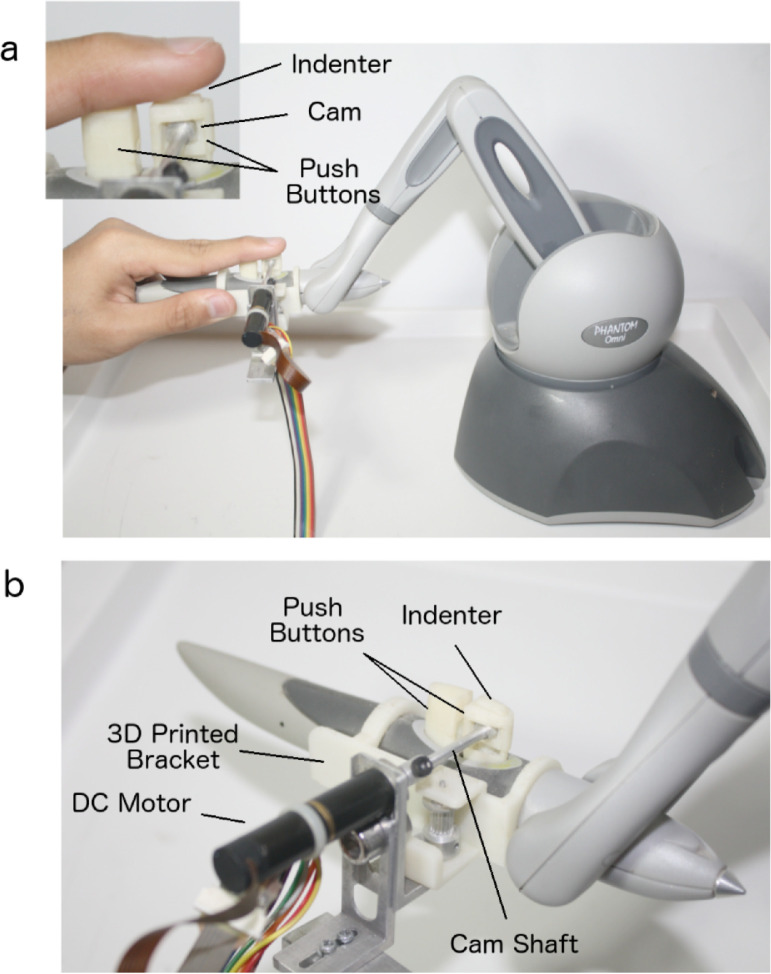
The single-button indenter that was used to provide the haptic feedback. a) The placement of the index finger on the indenter device. b) The components of the haptic device (Adapted from [2]).

A single-button indenter was considered to provide the haptic feedback on the fingertip [3]. The indenter was mounted on the stylus of Geomagic Touch Haptic Device (Fig. 7). The stylus contains two buttons. The indenter was designed to be fit with the front push button where the index finger can rest on. The indenter uses a cam and follower mechanism to convert the rotary motion of the DC motor to a vertical displacement motion. An encoder embedded inside the DC motor was used to determine the angular position, and hence, the corresponding vertical displacement of 0 to 2 mm. A control loop using PID controller was established using a LabView script to control the operations of the DC motor. More in-depth details about the experimental setup can be found in [2].

### 2.2 Perceptual experiments

**Fig. 8:**
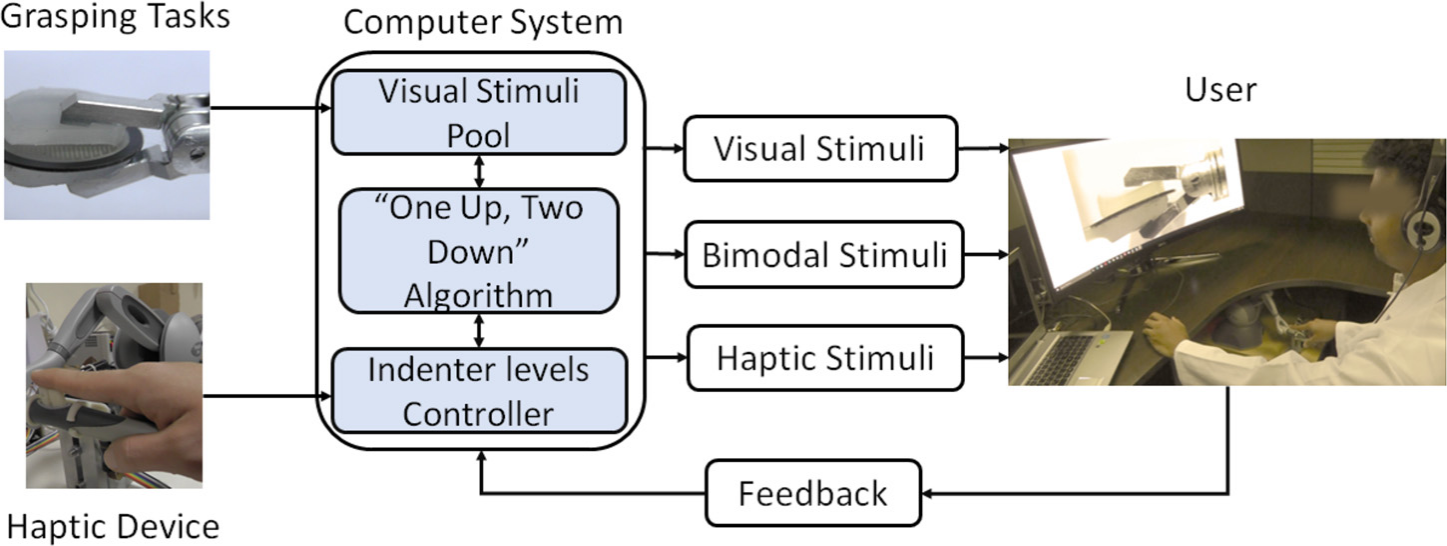
The perceptual experiments that were conducted to acquire the data. The user experiences three stimuli groups namely visual, haptic, and bimodal. The “one up, two down” algorithm controls the flow of stimuli presentations in every mode (Adapted from [2]).

The data were collected from sixty subjects (38 males and 22 females) performing the three experiments namely visual-alone, haptic-alone, and combined. An overview of the experiments is shown in Fig. 8. The descriptions for each experiment are provided in the next subsections.

### 2.3 Visual-alone stimuli

The visual stimuli involved presenting the user with a series of videos depicting the grasping of a soft tissue at different force levels. The considered grasping force range was from 0.05 N to 0.50 N. Ten videos were produced showing different grasping forces with a step of 0.05 N. During each iteration, two random presentations, but with certain force difference, were showen to the user. After two presentations, the user was asked: “Which of the two forces is higher?”. Then, the user was prompted to reply with either “First” or “Second”. The force difference between two consecutive presentations was changing depending on the user's reponses. The flow of the presentations relied on the “one up, two down” algorithm till the termination conditions were met. The visual-alone stimuli was displayed using a 35” LCD. Further details can be found in [2].

### 2.4 Haptic-alone stimuli

This type of stimuli involved applying an indentation at the user's fingertip using the haptic device. The indentation range was from 0 to 2 mm at 0.2 mm displacement. The device was placed near the right hand of the user. A headphone playing white noise was used to filter any ambient sounds including the noise emitted by the haptic device. Similar to visual-alone, ten different indentation levels were considered. The participants were then asked: “Which of the two forces is higher?” after the execution of two indentations. The flow of the experiments was based on the same algorthim described previously. Further details can be found in [2].

### 2.5 Bimodal stimuli

In this experiment, both the haptic and visual stimuli were combined. The timing and execution of both stimuli were matched and synchronized. The displacements of the indenter from 0 to 2 mm were made to correspond with grasping force values of 0.05 to 0.50 N. Similar to the visual-alone and haptic-alone, the bimodal experiment executed two consecutive presentations then acquired the user's response.

**Algorithm**

**Fig. 9:**
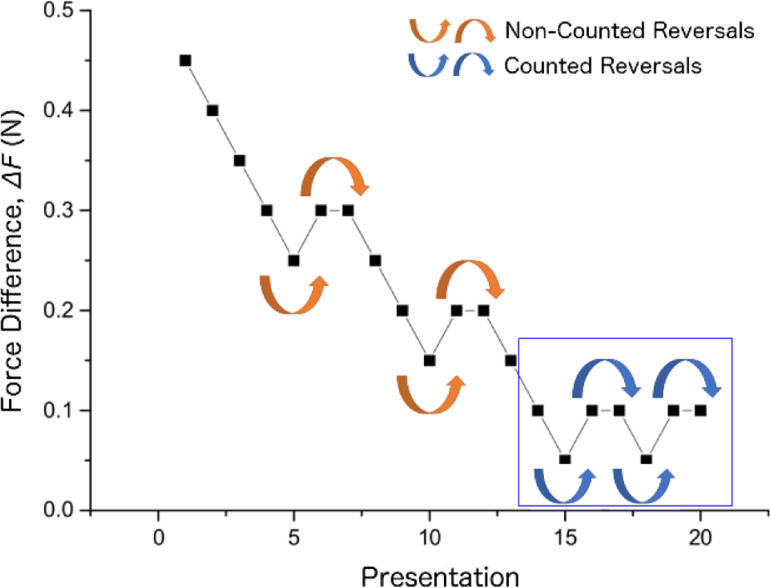
An example demonstrating “one up, two down” algorithm that was used to caluculate the threshold forces for one of the participants. The algorthim has terminated after detecting a total of 8 reversals indicated by the arc arrows. The force difference values at the last four reversals (i.e. inside the box) were used in calculating the threshold force difference for this participant by considering the average value. For example, this participant achieved a mean threshold force value of 0.075 N and the experiment was terminated at presentation (iteration) set 20 (Adapted from [2]).

The adaptive “one up, two down” algorithm was considered to determine the smallest perceived force difference. The algorthim controlled the execution of the presentations in the three perceptual experiments by traking the responses of the participants. At the start of an experiment, the algorthim set the force difference between the two presentations to the maximum value (i.e., 0.45 N) and decremented gradually (i.e., by 0.05 N) as the user provides the correct answers. However, once the user responses incorrectly, this force difference is increamented (i.e., by 0.05 N) and requires two concesective correct answers to decrement again. This transition is referred to as a “*reversal*” and was used as a terminating condition (i.e., total of 8 reversals) along with the total number of iterations (i.e., 50) for the experiments. Additionally, it was considered in the calculation of the threshold force difference by considering the last four reversals (Fig. 9).

## Ethics Statement

An informed consent was obtained from each participant. The procedures did not include invasive or potentially dangerous methods and were in accordance with the Code of Ethics of the World Medical Association (Declaration of Helsinki).

## CRediT Author Statement

**John-John Cabibihan:** Formal analysis, Conceptualization, Funding acquisition, Writing – review & editing; **Ahmad Yaser Alhaddad:** Methodology, Investigation, Data curation, Writing – original draft; **Tauseef Gulrez:** Methodology, Software, Writing – review & editing; **W. Jong Yoon:** Conceptualization, Supervision, Validation, Writing – review & editing.

## Declaration of Competing Interest

The authors declare that they have no known competing financial interests or personal relationships which have, or could be perceived to have, influenced the work reported in this article.

## References

[1] Cabibihan J.-J., Alhaddad A.Y., Gulrez T., Yoon W.J. (2021). https://dataverse.harvard.edu/dataset.xhtml?persistentId=doi:10.7910/DVN/QGKEUW.

[2] Cabibihan J.-J., Alhaddad A.Y., Gulrez T., Yoon W.J. (July 2021). Influence of visual and haptic feedback on the detection of threshold forces in a surgical grasping task. IEEE Robot. Autom. Lett..

[3] Gulrez T., Yoon W.J. (2018). https://patents.google.com/patent/US9946350B2/en.

